# Valedictory Address

**Published:** 1853-04

**Authors:** 


*Valedictory Address,* by JOSEPH CARSON, M. D., Professor of Materia-Medica and Pharmacy, *before the Graduating Class in the University of Pennsylvania, April* 2, 1853.

THIS address of Dr. Carson's is well worthy a perusal, as it is replete with noble sentiments, uttering with emphasis the claims of the profession upon each and all of its members, young, as well as old, those who are just launching their barks, as well as those who have been long veterans in active service. And that these claims are not only professional in upholding the dignity, and advancing the progress of medicine, but that they are also social and embody all the better feelings of our nature, in behalf of suffering humanity.

